# Editorial: Advances in mechanically bonded molecules

**DOI:** 10.3389/fchem.2022.1095082

**Published:** 2022-12-06

**Authors:** Carson J. Bruns, Wei Wang, Keiji Hirose

**Affiliations:** ^1^ University of Colorado Boulder, Boulder, CO, United States; ^2^ East China Normal University, Shanghai, China; ^3^ Osaka University, Osaka, Japan

**Keywords:** rotaxane, catenane, polyrotaxane, mechanical bond, stereochemistry, molecular machines, molecular shuttles

## Introduction

Mechanically bonded molecules such as rotaxanes and catenanes are utilized increasingly in energy storage, (Choi et al., 2017) bio-interfaces, (Arisaka and Yui, 2019) advanced functional materials (Mena-Hernando and Pérez, 2019) and molecular machines (Bruns and Stoddart, 2016) (among many others), motivating continued research on both the fundamental and applied science of mechanical bonds. The *Frontiers in Chemistry* topic collection on Advances in Mechanically Bonded Molecules is dedicated to progress on the fundamental science of how novel structures and dynamics emerge from molecular entanglements.

## Progress on mechanically bonded structures

*Radial [n]catenanes.* Wu et al. report a new synthesis of [3]- and [4]catenanes by way of coordination-driven self-assembly of carboxylated Cu(I) metallo-pseudo [2]rotaxanes with divalent organoplatinum complexes by [2 + 2] or [3 + 3] macrocyclization. Although topologically equivalent assemblies have long been accessed by metal-ligand complexation (Whang et al., 1998) the team’s use of Pt–O bonds to form charge-neutral central rings expands the toolbox of motifs available for synthesizing radial [*n*]catenanes.

*Pillar[5]arene [1]rotaxanes.* In a [1]rotaxane, the macrocyclic moiety is bound both chemically and mechanically to the corresponding axle. Combining six stopper precursors with two pseudo [1]rotaxane precursors, Ma et al. generated a library of a dozen new [1]rotaxanes based on a well-known (Strutt et al., 2011) alkylamine-threaded pillar [5]arene (P5A) motif. The precursors’ terminal amines react with pentanedione, formylazobenzene, or salicylaldehyde derivatives with up to 89% efficiency, widening the scope of stoppering chemistry for P5A mechanomolecules.

*Polyrotaxane networks.* First introduced in 2001 by (Ito and Okumura, 2001), polyrotaxane gels exhibit remarkable elasticity, attributed to a “pulley effect” whereby mechanically bonded sliding crosslinks dissipate stress. (Hart et al., 2021). Despite hundreds of papers on slide-ring gels of α-cyclodextrin-threaded poly (ethylene glycol), the kinetics of cross-linking in this system were not reported until now. By varying reactant concentrations and temperature while monitoring the evolution of viscoelastic moduli *in situ*, Dikshit and Bruns determine the rate law and activation energy for elastically effective cross-linking. Of practical experimental use, the kinetics data also unlock a new way to fine-tune viscoelastic moduli by timed quenching of the cross-linking reaction.

## Progress on mechanomolecular dynamics

*Ring-over-ring deslipping.* Unlike ring shuttling and circumrotation motions commonly observed in rotaxanes, ring-over-ring translation (through-the-annulus site exchange of two rings encircling the same axle) is rarely demonstrated. (Zhu et al., 2018). Hoshino et al. observed this rare phenomenon when the central cyclophane of a hetero (Bruns and Stoddart, 2016) rotaxane dissociated from the dumbbell by passing over its mechanically bonded [24]crown-8 rings near the stoppers. This ring-through-ring process diversifies the types of mechanical motions rotaxane-based molecular machines can perform.

*Switchable mechanical chirality.* Ishiwari and Takata describe a simple [2]rotaxane switch with sophisticated stereochemical dynamics. The switch is prochiral when its asymmetric crown ether encircles the central dibenzylammonium (DBA) unit of a symmetrical axle, but it exhibits mechanically planar chirality when alkylation or deprotonation of DBA displaces the ring from center. The resulting left- and right-handed enantiomers interconvert as the ring shuttles between two sides of the dumbbell. This “mechanostereoinversion” slows down as the steric bulk of the *N*-alkyl group increases, allowing enantiomeric resolution by chiral HPLC in some cases. This work lays a strong foundation for molecular machines that switch between optically active and inactive states.

*Photodriven molecular shuttles*. Switchable molecular shuttles are a canonical archetype of artificial molecular machines (AMMs). Since light is among the most attractive fuel sources (by virtue of convenience, speed, and minimal waste), photo-driven molecular shuttles represent one of the largest classes of AMMs. A review by Yao et al. summarizes recent progress on molecular shuttles driven by light, highlighting technological applications and prospects for future development.

*Rigidity and flexibility in rotaxanes*. In a topic not previously reviewed, Fadler and Flood explore the role of flexibility and rigidity on the properties of rotaxanes. Threading rings onto axles leads to their mutual rigidification, the extent of which is highly system-specific, yet it impacts the energy barriers and timescales of mechanical motion and even bulk material properties. These concepts can be leveraged in the design of rotaxanes with highly tailored functions.

Since bonding is the foundation of molecular structure and dynamics, mechanical bonds on the molecular scale profoundly impact material properties. The field remains rich in untapped potential for new discoveries, and will no doubt continue to fuel technological progress as the fundamental science of mechanical bonds continues to be nurtured.
